# Supplementary material for: Optimization of rice panicle architecture by specifically suppressing ligand–receptor pairs
Source: Nat Commun. 2023 Mar 24;14:1640. doi: 10.1038/s41467-023-37326-x (PMC10039049; doi:10.1038/s41467-023-37326-x)
Supplement: Supplementary file 1 — Supplementary Information [file 41467_2023_37326_MOESM1_ESM.pdf]

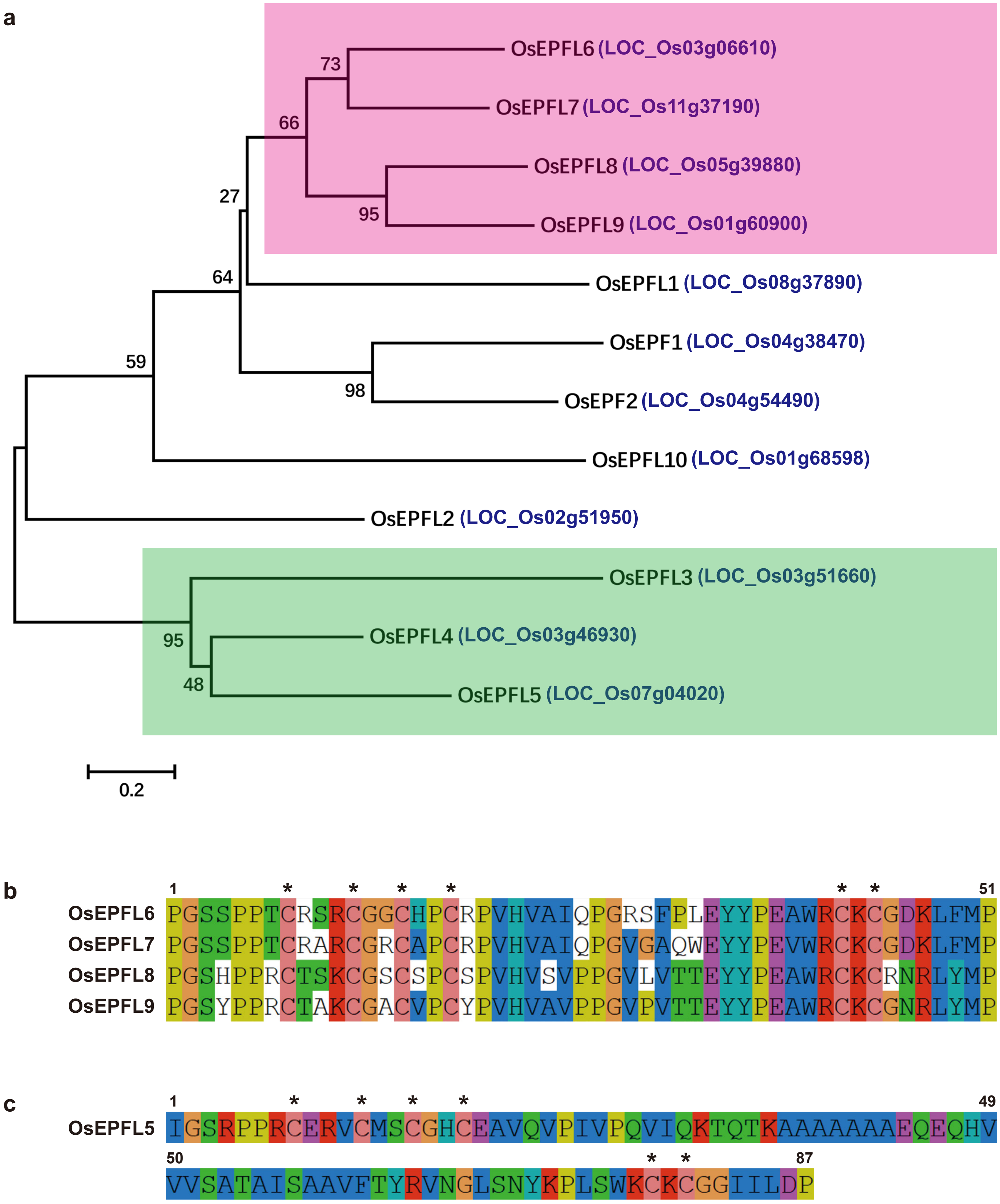

Supplementary Fig. 1 Phylogenetic tree of the rice *OsEPF/OsEPFL* gene family and comparison of the cysteine-rich mature peptides.
a, Phylogenetic tree constructed using MEGA 6. The evolutionary history was inferred using the Neighbor-Joining method. Evolutionary distances were computed using the JTT matrix-based method using the number of amino acid substitutions per site. Where values were ≥ 50%, the percentage of replicate trees where the associated taxa clustered together in the bootstrap test (1000 replicates) are shown close to each branch. The tree was drawn to scale, with branch lengths in the same units as those of the evolutionary distances used to infer the phylogenetic tree. **b**, Amino acid sequence alignment of the predicted mature small peptides OsEPFL6, OsEPFL7, OsEPFL8, and OsEPFL9. **c**, Amino acid sequence of predicted mature small peptide OsEPFL5. Alignment was conducted with ClustalX. Asterisks indicate cysteine residues.

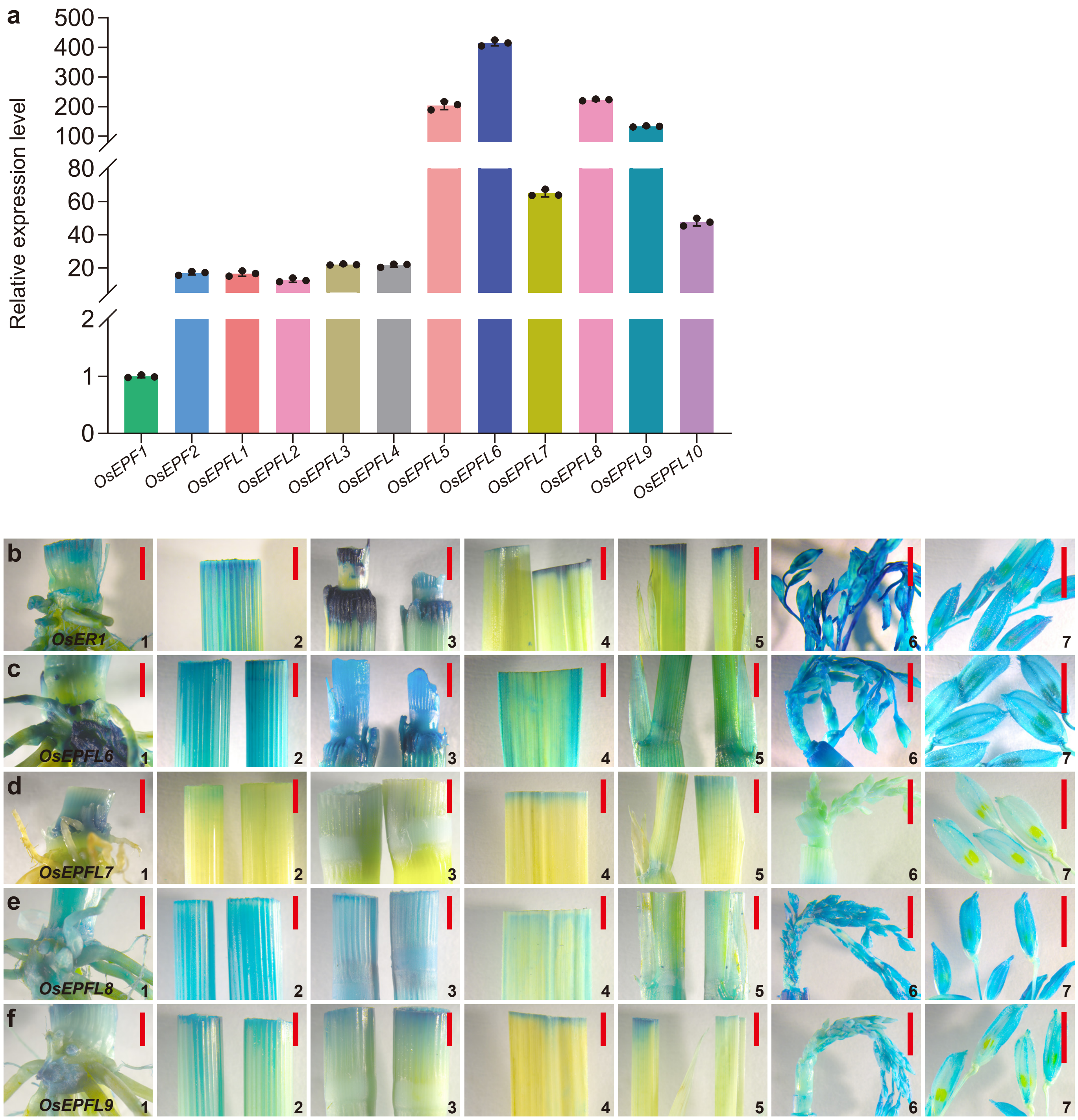

Supplementary Fig. 2 Expression pattern of the *OsEPF/OsEPFL* gene family members and GUS staining analysis of *OsER1*, *OsEPFL6*, *OsEPFL7*, *OsEPFL8*, and *OsEPFL9*.
a, Comparison of the relative expression levels of *OsEPF1*, *OsEPF2*, *OsEPFL1*, *OsEPFL2*, *OsEPFL3*, *OsEPFL4*, *OsEPFL5*, *OsEPFL6*, *OsEPFL7*, *OsEPFL8*, *OsEPFL9*, and *OsEPFL10* in young panicles (n = 3 pooled tissues, three young panicles per pool). *OsUBQ5* was used as the internal reference gene to normalize expression data. Values are given as the mean ± SD. **b-f**, GUS staining of *OsER1* (**b**), *OsEPFL6* (**c**), *OsEPFL7* (**d**), *OsEPFL8* (**e**), and *OsEPFL9* (**f**). 1, root; 2, culm internode; 3, culm node; 4, leaf blade; 5, leaf sheath; 6, young panicles; 7, spikelet hulls. Scale bars = 1 cm. The source data underlying the statistical analysis in **a** are provided in the Source Data file.

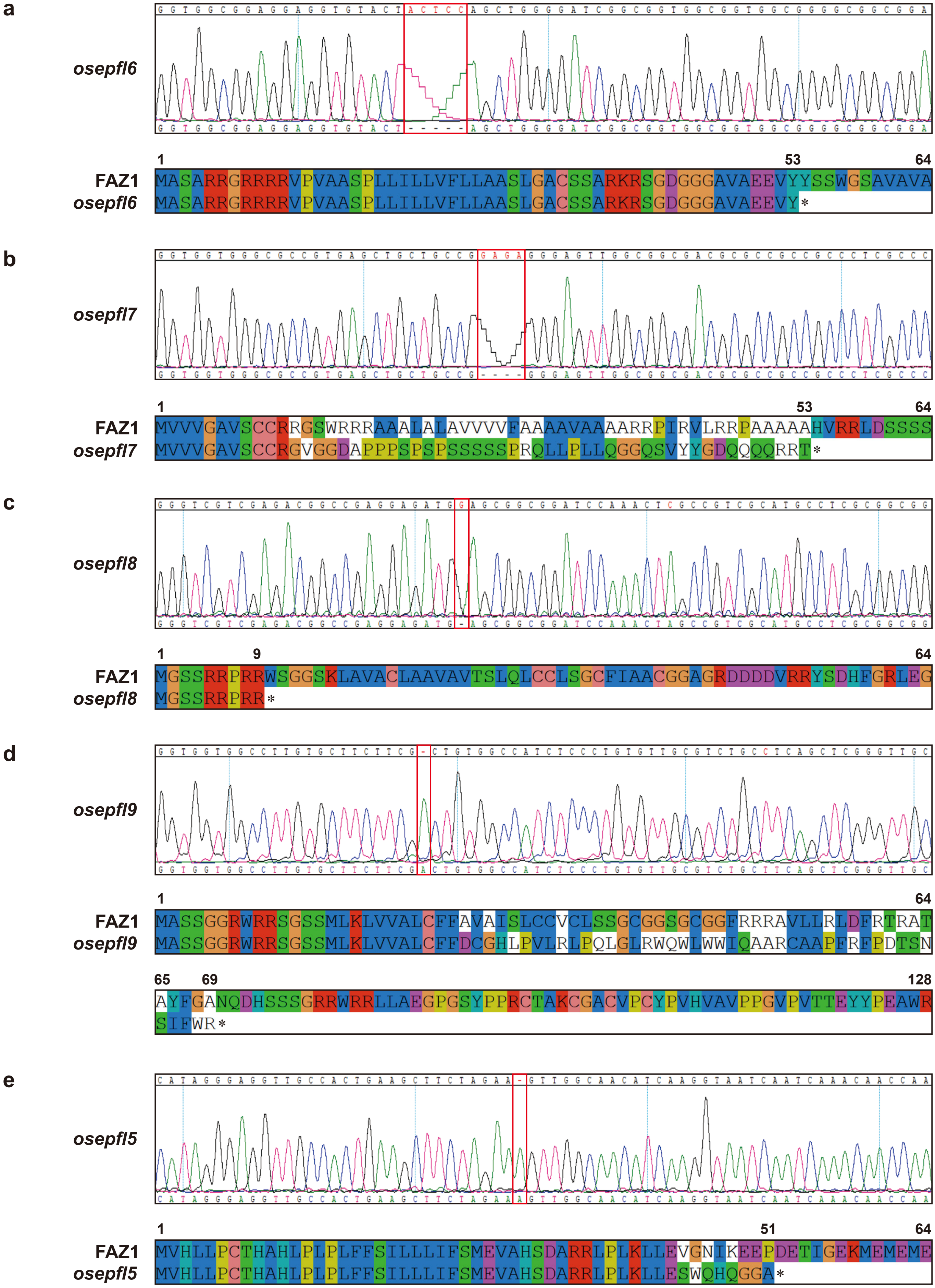

Supplementary Fig. 3 Genotyping of *osepfl6*, *osepfl7*, *osepfl8*, *osepfl9*, and *osepfl5* null mutations.

a-e, Identification of the *osepfl6* (**a**), *osepfl7* (**b**), *osepfl8* (**c**), *osepfl9* (**d**), and *osepfl5* (**e**) null mutations by sequencing and alignment of the mutant sequences with the corresponding FAZ1 sequences. Red boxes indicate a deletion or insertion mutation causing premature termination. Asterisks indicate the positions of premature termination.

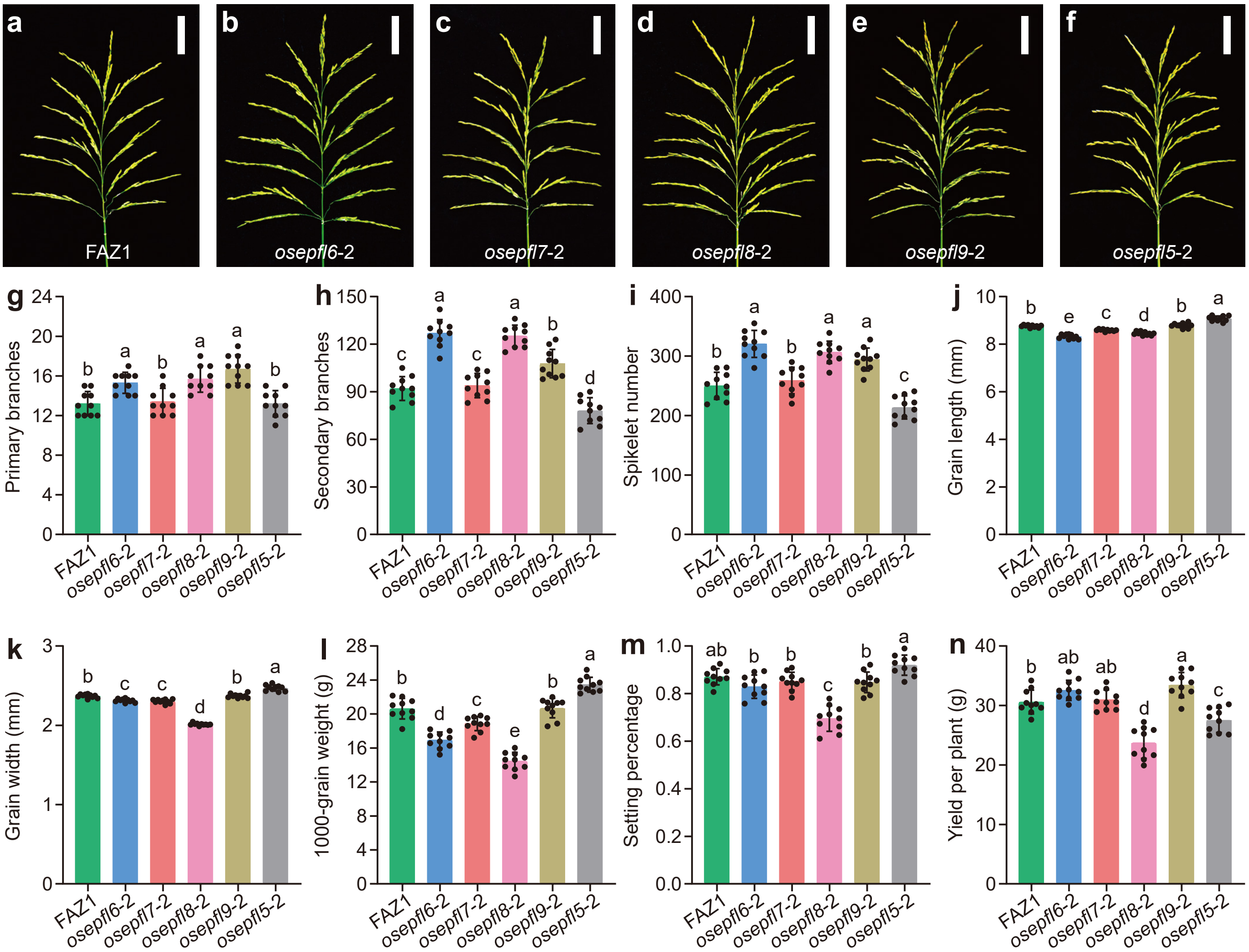

Supplementary Fig. 4 Phenotyping of the additional alleles of *osepfl6*, *osepfl7*, *osepfl8*, *osepfl9*, and *osepfl5* null mutants.

a-f, Rice panicles from FAZ1 (**a**) and the *osepfl6*-2 (**b**), *osepfl7*-2 (**c**), *osepfl8*-2 (**d**), *osepfl9*-2 (**e**), and *osepfl5*-2 (**f**) mutants. Scale bar = 5 cm. **g-n**, Comparison of average number of primary branches (**g**), number of secondary branches (**h**), spikelet number per panicle (**i**), grain length (**j**), grain width (**k**), 1000-grain weight (**l**), setting percentage (**m**), and yield per plant (**n**) between FAZ1 and *osepfl6*-2, *osepfl7*-2, *osepfl8*-2, *osepfl9*-2, and *osepfl5*-2 mutants. Values are given as the mean ± SD (n = 10 plants). Different letters indicate statistical significance groups at $p < 0.05$ (one-way ANOVA with post-hoc Tukey's multiple comparison test). The source data underlying the statistical analysis in **g-n** are provided in the Source Data file.

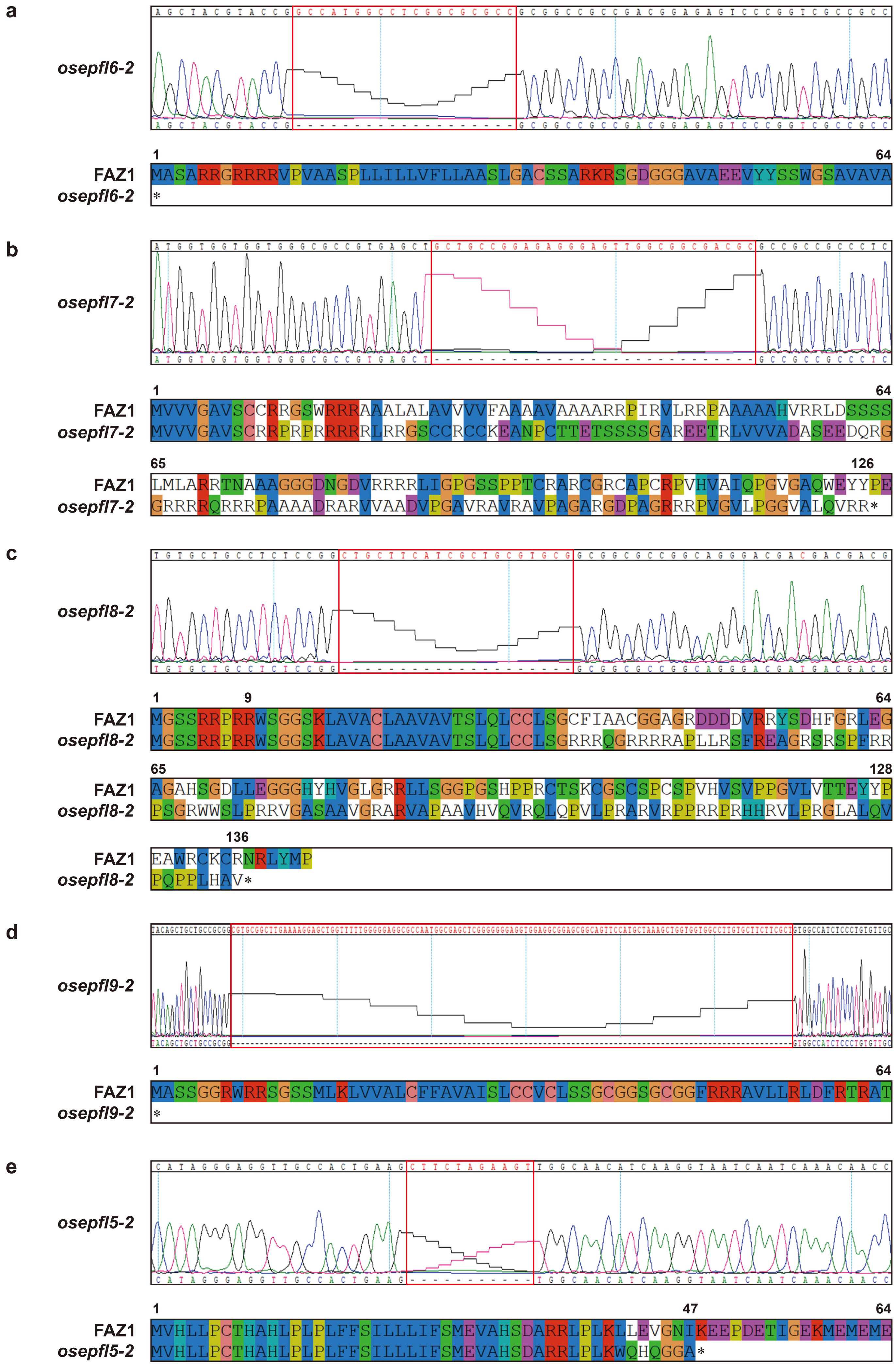
a
osepfl6-2
1
64
FAZ1 MASARRGRRRRVPVAASPLLILLVFLLAASLGACSSARKRSGDGGGAVAEEVYYSSWGSAVAVA
osepfl6-2 *
b
osepfl7-2
1
64
FAZ1 MVVVGAVSCCRRGSWRRRAAALALAVVVVFAAAAVAAAARRPIRVLRRPAAAAAHVRRLDSSSS
osepfl7-2 MVVVGAVSCRRPRPRRRRRLRRGSCCRCCKEANPCTTETSSSSGAREETRLVVVADASEEDQRG
65
126
FAZ1 LMLARRTNAAAGGGDNGDVRRRRLIGPGSSPPTCRARCGRCAPCRPVHVAIQPGVGAQWEYYPE
osepfl7-2 GRRRRQRRRPAAAADRARVVAADVPGAVRAVRAVPAGARGDPAGRRRPVGVLPGGVALQVRR*
c
osepfl8-2
1
9
64
FAZ1 MGSSRRPRRWSGGSKLAVACLAAVAVTSLQLCCLSGCFIAACGGAGRDDDDVRRYSDHFGRLEG
osepfl8-2 MGSSRRPRRWSGGSKLAVACLAAVAVTSLQLCCLSGRRRQGRRRRAPLLRSFREAGRSRSPFRR
65
128
FAZ1 AGAHSGDLLEGGGHYHVGLGRRLLSGGPGSHPPRCTSKCGSCSPCSPVHVSVPPGVLVTTEYYP
osepfl8-2 PSGRWWSLPRRVGASAAVGRARVAPAAVHVQVRQLQPVLPRARVRPPRRPRHHRVLPRGLALQV
136
FAZ1 EAWRCKCRNRLYMP
osepfl8-2 PQPPLHAV*
d
osepfl9-2
1
64
FAZ1 MASSGGRWRRSGSSMLKLVVALCFFAVAISLCCVCLSSGCGGSGCGGFRRRAVLLRLDFRTRAT
osepfl9-2 *
e
osepfl5-2
1
47
64
FAZ1 MVHLLPCTHAHLPLPLFFSILLLIFSMEVAHSDARRLPLKLLEVGNIKEEPDETIGEKMEMEME
osepfl5-2 MVHLLPCTHAHLPLPLFFSILLLIFSMEVAHSDARRLPLKWQHQGGA*

Supplementary Fig. 5 Genotyping of *osepfl6*-2, *osepfl7*-2, *osepfl8*-2, *osepfl9*-2, and *osepfl5*-2 null mutations.

a-e, Identification of the *osepfl6*-2 (**a**), *osepfl7*-2 (**b**), *osepfl8*-2 (**c**), *osepfl9*-2 (**d**), and *osepfl5*-2 (**e**) null mutations by sequencing and alignment of the mutant sequences with the corresponding FAZ1 sequences. Red boxes indicate a deletion mutation causing premature termination. Asterisks indicate the positions of premature termination.

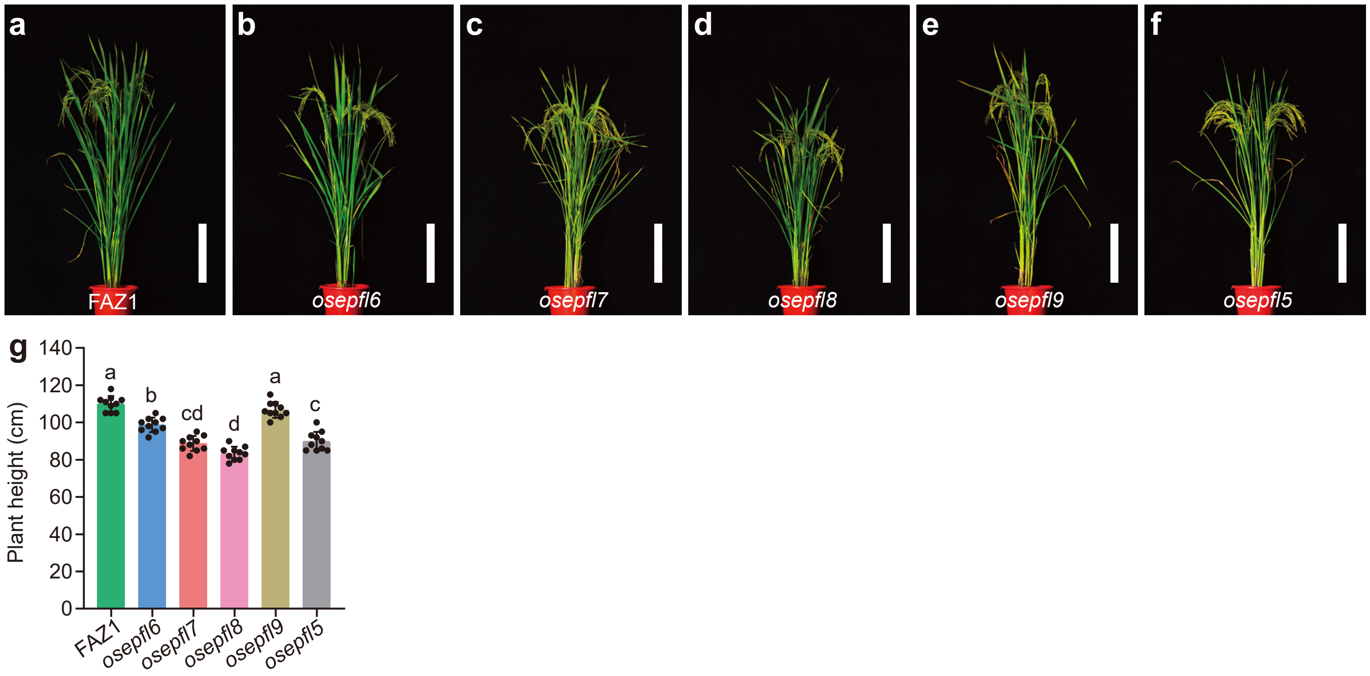

Supplementary Fig. 6 *OsEPFL6*, *OsEPFL7*, *OsEPFL8*, and *OsEPFL5* are associated with plant architecture in rice.

a-f, Plant architecture of FAZ1 (**a**) and the *osepfl6* (**b**), *osepfl7* (**c**), *osepfl8* (**d**), *osepfl9* (**e**), and *osepfl5* (**f**) mutants. Scale bar = 20 cm. **g**, Comparison of average plant height between FAZ1, *osepfl6*, *osepfl7*, *osepfl8*, *osepfl9*, and *osepfl5*. Values are given as the mean ± SD (n = 10 plants). Different letters indicate statistical significance groups at $p < 0.05$ (one-way ANOVA with post-hoc Tukey's multiple comparison test). The source data underlying the statistical analysis in **g** are provided in the Source Data file.

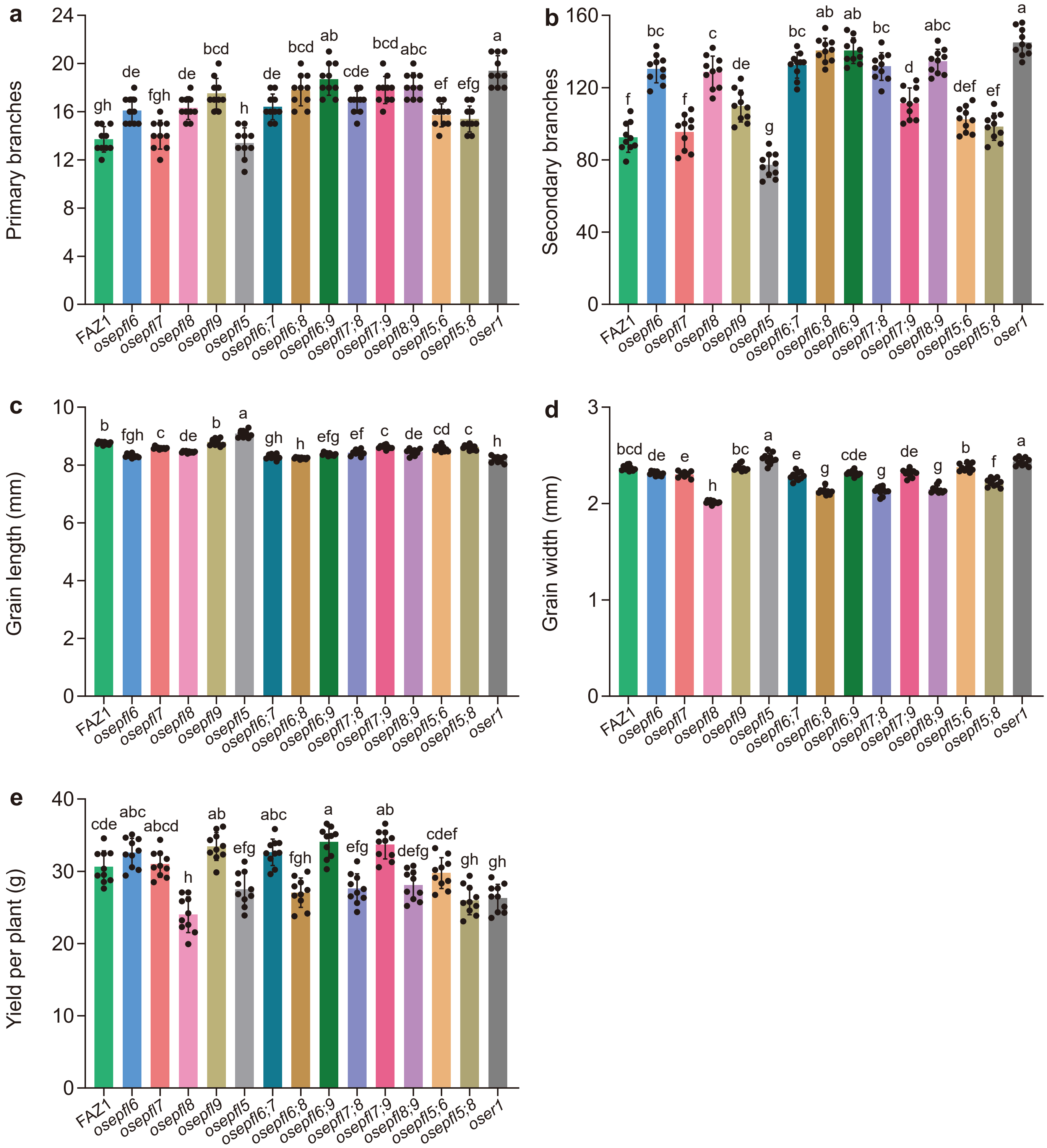

Supplementary Fig. 7 Comparison of yield traits of *OsEPFL6*, *OsEPFL7*, *OsEPFL8*, *OsEPFL9*, and *OsEPFL5* double mutants.

a-e, Comparison of average number of primary branches (**a**), number of secondary branches (**b**), grain length (**c**), grain width (**d**), and yield per plant (**e**) between FAZ1, *osepfl6*, *osepfl7*, *osepfl8*, *osepfl9*, *osepfl5*, *osepfl6;7*, *osepfl6;8*, *osepfl6;9*, *osepfl7;8*, *osepfl7;9*, *osepfl8;9*, *osepfl5;6*, *osepfl5;8*, and *oser1*. Values are given as the mean ± SD (n = 10 plants). Different letters indicate statistical significance groups at $p < 0.05$ (one-way ANOVA with post-hoc Tukey's multiple comparison test). The source data underlying the statistical analysis in **a-e** are provided in the Source Data file.

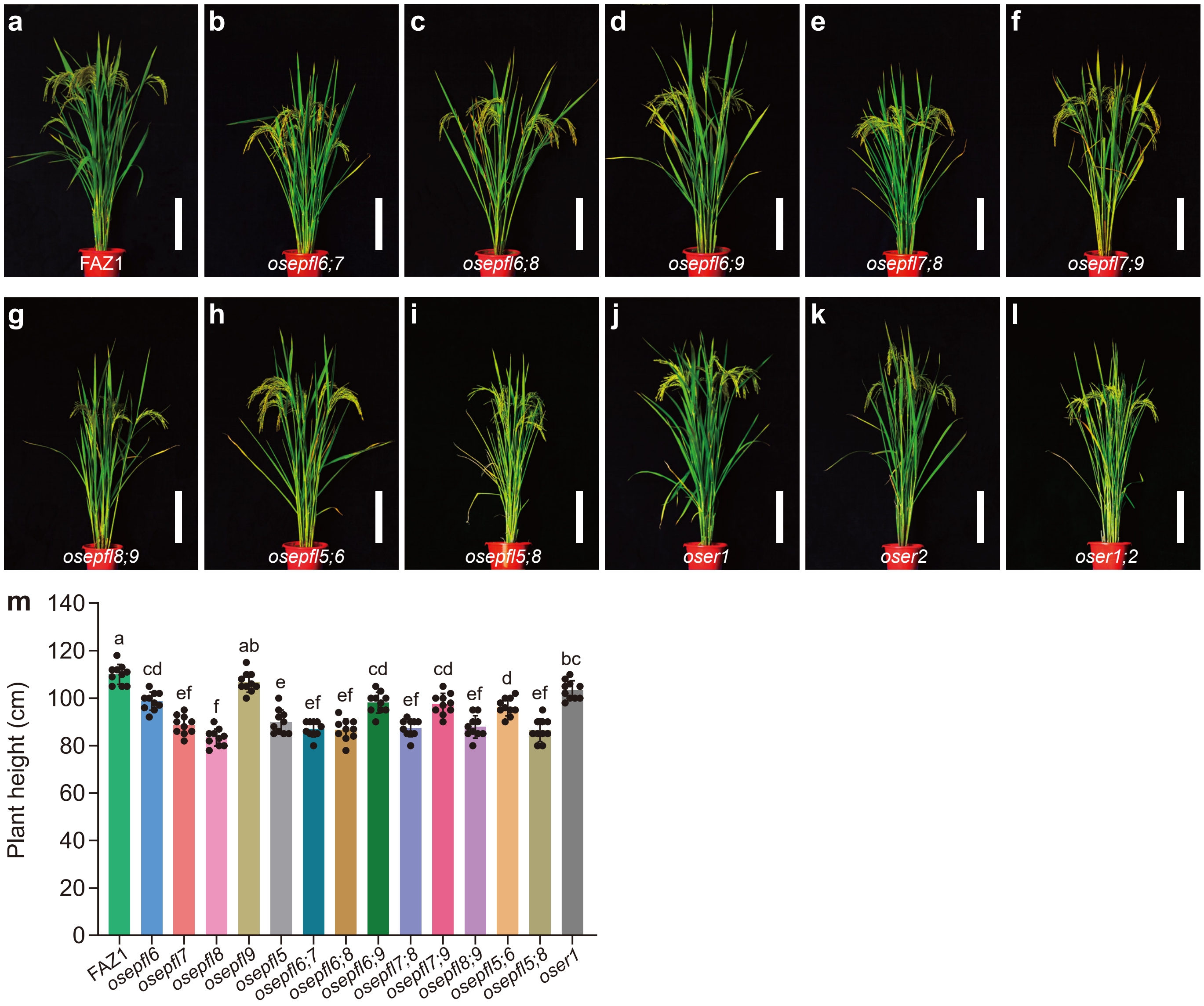

Supplementary Fig. 8 Plant architecture of *OsEPFL6*, *OsEPFL7*, *OsEPFL8*, *OsEPFL9*, and *OsEPFL5* double mutants.

a-l, Plant architecture of FAZ1 (**a**) and the *osepfl6;7* (**b**), *osepfl6;8* (**c**), *osepfl6;9* (**d**), *osepfl7;8* (**e**), *osepfl7;9* (**f**), *osepfl8;9* (**g**), *osepfl5;6* (**h**), *osepfl5;8* (**i**), *oser1* (**j**), *oser2* (**k**), and *oser1;2* (**l**) mutants. Scale bar = 20 cm. m, Comparison of average height between FAZ1, *osepfl6*, *osepfl7*, *osepfl8*, *osepfl9*, *osepfl5*, *osepfl6;7*, *osepfl6;8*, *osepfl6;9*, *osepfl7;8*, *osepfl7;9*, *osepfl8;9*, *osep-fl5;6*, *osepfl5;8*, and *oser1*. Values are given as the mean ± SD (n = 10 plants). Different letters indicate statistical significance groups at $p < 0.05$ (one-way ANOVA with post-hoc Tukey's multiple comparison test). The source data underlying the statistical analysis in **m** are provided in the Source Data file.

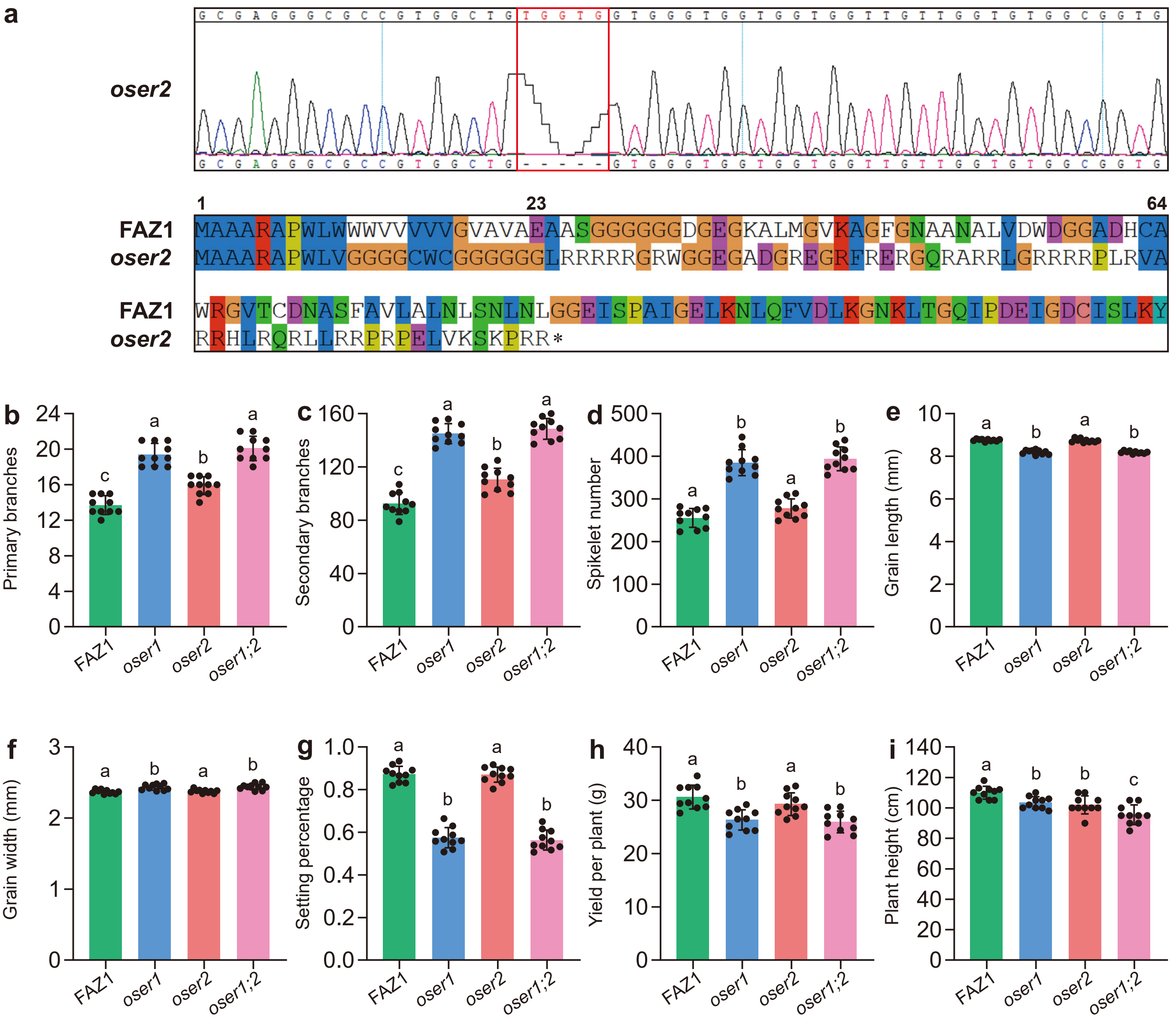

Supplementary Fig. 9 *OsER1* predominantly contributes to rice panicle morphogenesis. **a**, Identification of the *oser2* mutation by sequencing and alignment of mutant sequences with the FAZ1 sequence. Red box indicates a deletion mutation causing premature termination. Asterisk indicates the positions of premature termination. **b-i**, Comparison of average number of primary branches (**b**), number of secondary branches (**c**), spikelet number per panicle (**d**), grain length (**e**), grain width (**f**), setting percentage (**g**), yield per plant (**h**), and plant height (**i**) between FAZ1, *oser1*, *oser2*, and *oser1;2* plants. Values are given as the mean ± SD (n = 10 plants). Different letters indicate statistical significance groups at $p < 0.05$ (one-way ANOVA with post-hoc Tukey's multiple comparison test). The source data underlying the statistical analysis in **b-i** are provided in the Source Data file.

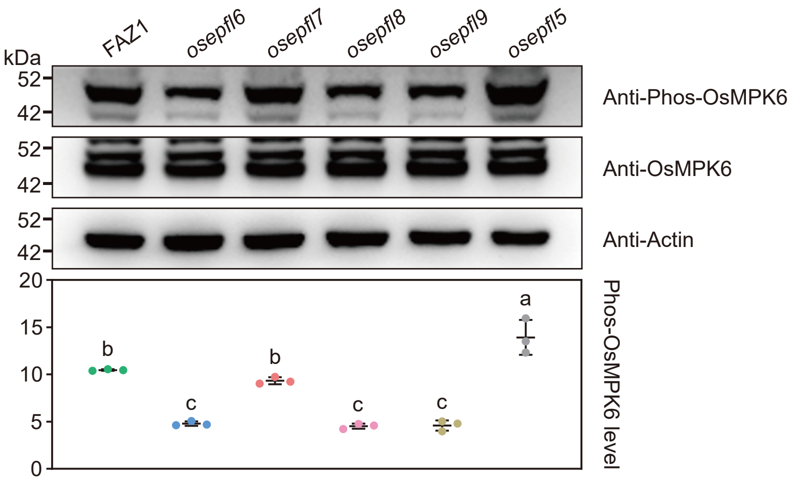

Supplementary Fig. 10 Loss of *OsEPFL6*, *OsEPFL7*, *OsEPFL8*, or *OsEPFL9* function diminished OsMPK6 phosphorylation level, but loss of *OsEPFL5* function elevated OsMPK6 phosphorylation level.

Comparison of OsMPK6 phosphorylation levels in FAZ1, *osepfl6*, *osepfl7*, *osepfl8*, *osepfl9*, and *osepfl5*. Proteins extracted from young panicles were analyzed with immunoblot using anti-Phos-OsMPK6 and anti-OsMPK6 antibodies. Anti-Actin antibody was used as the loading control. The graph shows the qualification of the relative levels of the phosphorylated OsMPK6. Values are given as the mean ± SD (n = 3 biological replicates). Different letters indicate statistical significance groups at $p < 0.05$ (one-way ANOVA with post-hoc Tukey's multiple comparison test). The source data underlying the uncropped image and statistical analysis are provided in the Source Data file.

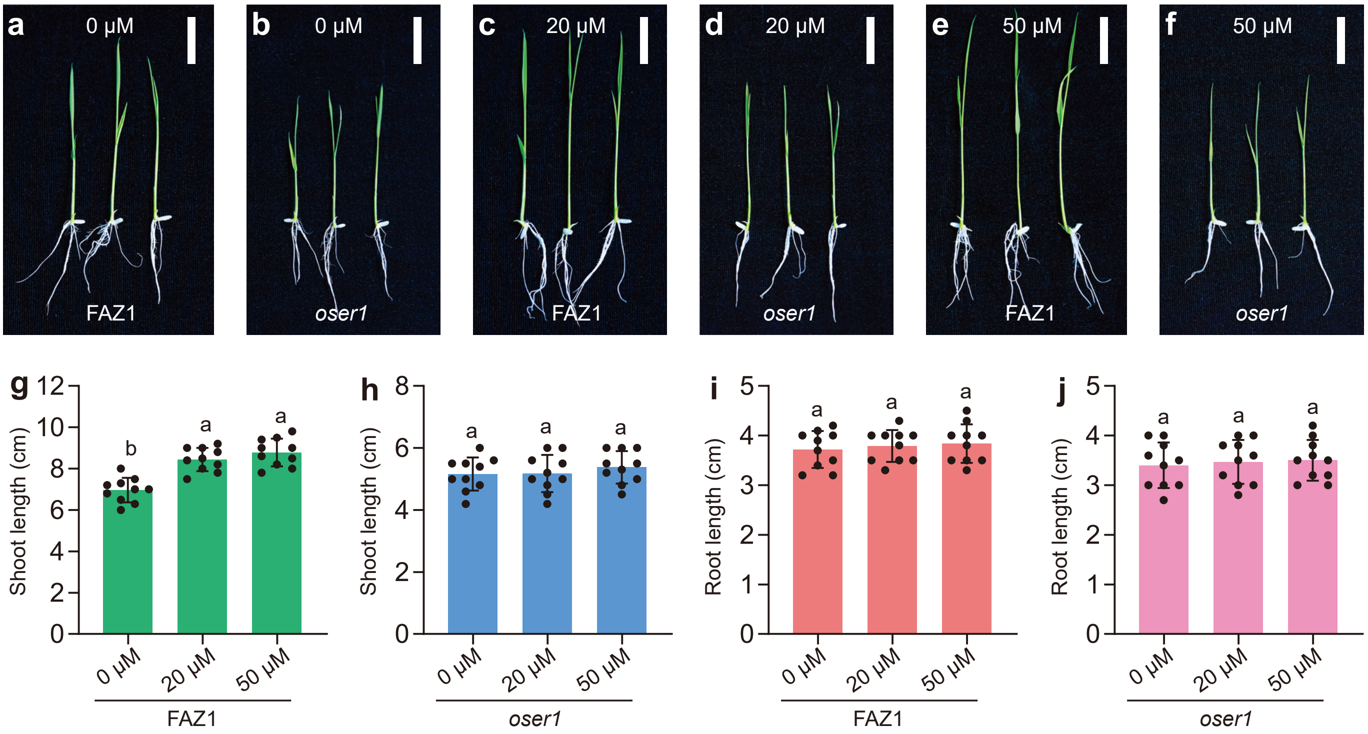

Supplementary Fig. 11 The mature OsEPFL6 small peptide promotes shoot elongation depending on *OsER1* in rice.

a-b, FAZ1 (**a**) and *oser1* seedlings (**b**) without OsEPFL6 small peptide treatment. **c-d**, FAZ1 (**c**) and *oser1* seedlings (**d**) treated with 20 μM OsEPFL6 small peptide. **e-f**, FAZ1 (**e**) and *oser1* seedlings (**f**) treated with 50 μM OsEPFL6 small peptide. Scale bar = 2 cm. Shoot and root length were measured after culturing seeds for 7 d in 1/2x MS medium with or without OsEPFL6 small peptide. **g-h**, Comparison of average shoot length in FAZ1 (**g**) and *oser1* plants (**h**) treated with 20 μM or 50 μM OsEPFL6 small peptide. **i-j**, Comparison of average root length in FAZ1 (**i**) and *oser1* plants (**j**) treated with 20 μM or 50 μM OsEPFL6 small peptide. Values are given as the mean ± SD (n = 10 plants). Different letters indicate statistical significance groups at $p < 0.05$ (one-way ANOVA with post-hoc Tukey's multiple comparison test). The source data underlying the statistical analysis in **g-j** are provided in the Source Data file.

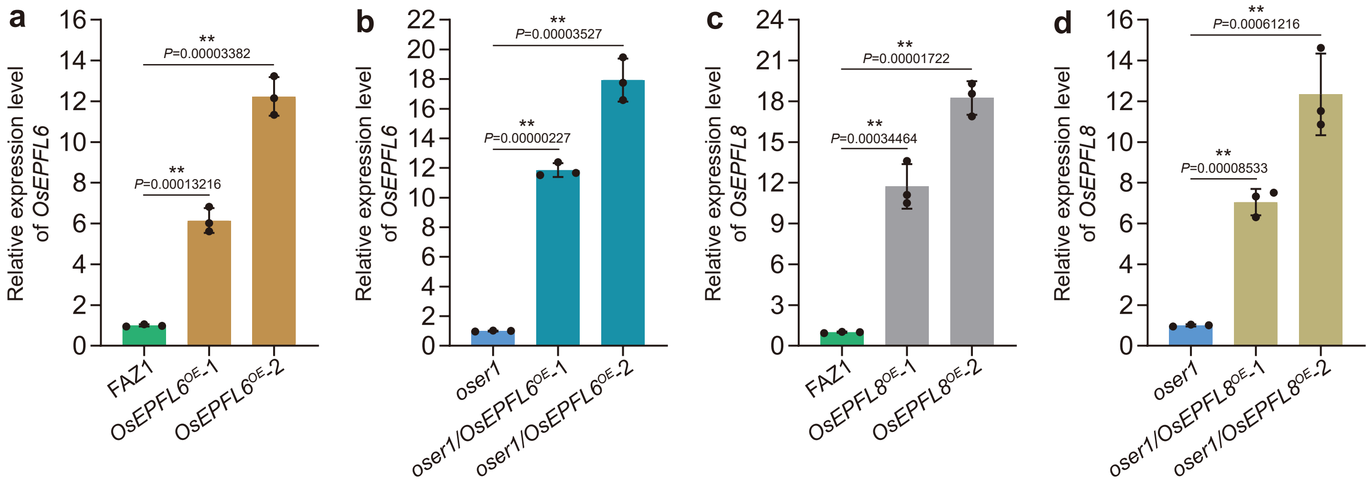

Supplementary Fig. 12 Confirmation of transgenic rice constitutively overexpressing *OsEPFL6* or *OsEPFL8*.

a-b, Relative expression levels of *OsEPFL6* in plants overexpressing *OsEPFL6* in the FAZ1 (**a**) or *oser1* (**b**) background. **c-d**, Relative expression levels of *OsEPFL8* in plants overexpressing *OsEPFL8* in the FAZ1 (**c**) or *oser1* (**d**) background. *OsUBQ5* was used as the internal reference gene to normalize expression data. Values are given as the mean ± SD (n = 3 plants). $^{**}p < 0.01$ indicates significant difference compared with FAZ1 or *oser1* by two-sided Student's *t*-test. The source data underlying the statistical analysis in **a-d** are provided in the Source Data file.

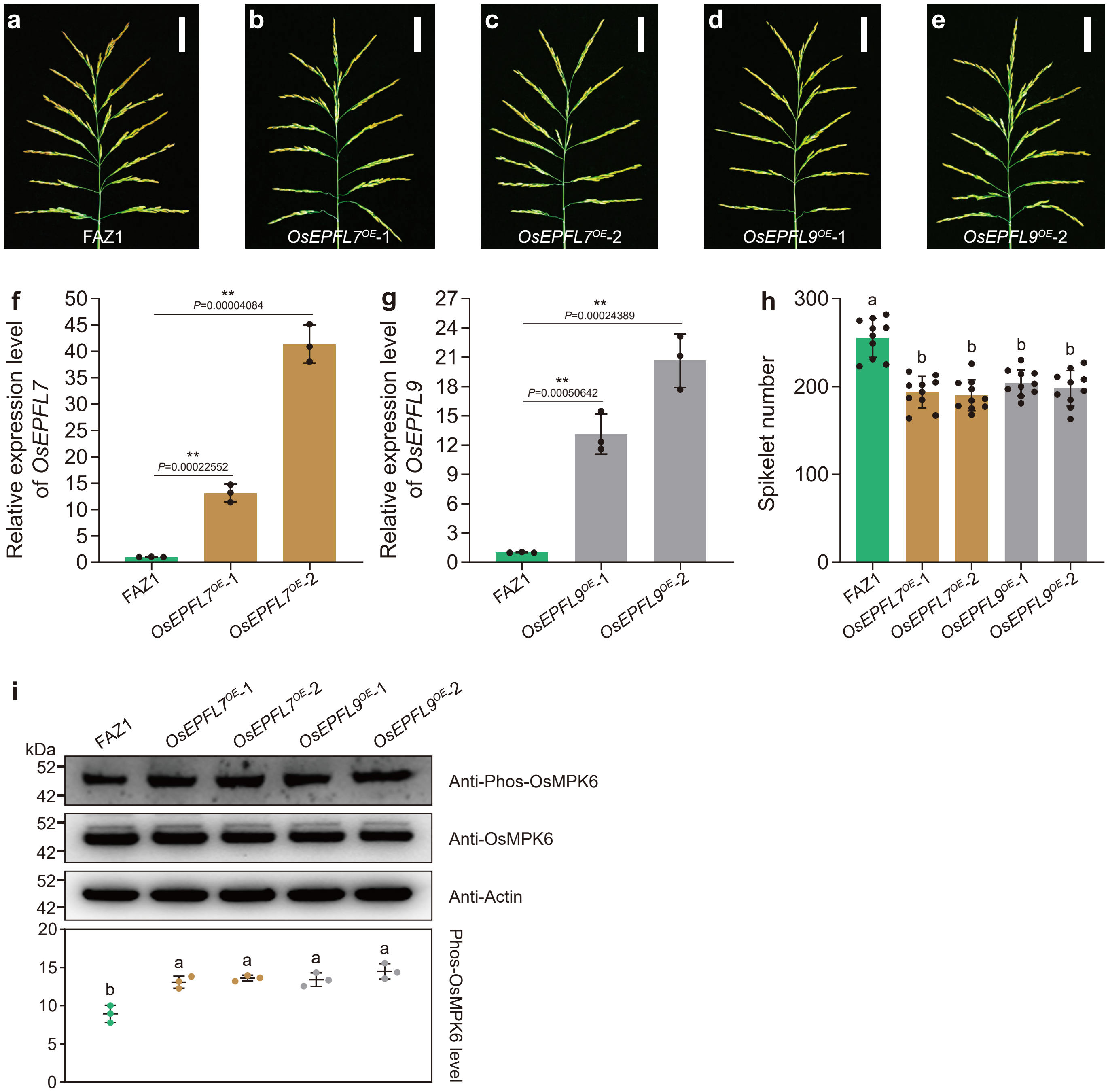

Supplementary Fig. 13 *OsEPFL7* and *OsEPFL9* negatively regulate spikelet number per panicle in rice.

a-e, Rice panicles from FAZ1 (**a**), the *OsEPFL7* overexpression lines *OsEPFL7*^{OE}-1 (**b**) and *OsEPFL7*^{OE}-2 (**c**), and the *OsEPFL9* overexpression lines *OsEPFL9*^{OE}-1 (**d**) and *OsEPFL9*^{OE}-2 (**e**) in the FAZ1 background. Scale bar = 5 cm. **f-g**, Relative expression levels of *OsEPFL7* (**f**) and *OsEPFL9* (**g**) in FAZ1 and overexpression lines. *OsUBQ5* was used as the internal reference gene to normalize expression data. Values are given as the mean ± SD (n = 3 plants). ***p* < 0.01 indicates significant difference compared with FAZ1 by two-sided Student's *t*-test. **h**, Comparison of average spikelet number per panicle between FAZ1, *OsEPFL7*^{OE}-1, *OsEPFL7*^{OE}-2, *OsEPFL9*^{OE}-1, and *OsEPFL9*^{OE}-2. Values are given as the mean ± SD (n = 10 plants). Different letters indicate statistical significance groups at $p < 0.05$ (one-way ANOVA with post-hoc Tukey's multiple comparison test). **i**, Comparison of OsMPK6 phosphorylation levels in FAZ1, *OsEPFL7*^{OE}-1, *OsEPFL7*^{OE}-2, *OsEPFL9*^{OE}-1, and *OsEPFL9*^{OE}-2. Proteins extracted from young panicles were analyzed via immunoblot with anti-Phos-OsMPK6 and anti-OsMPK6 antibodies. Anti-Actin antibody was used as the loading control. The graph shows the qualification of the relative levels of the phosphorylated OsMPK6. Values are given as the mean ± SD (n = 3 biological replicates). Different letters indicate statistical significance groups at $p < 0.05$ (one-way ANOVA with post-hoc Tukey's multiple comparison test). The source data underlying the statistical analysis in **f-i** and uncropped image in **i** are provided in the Source Data file.

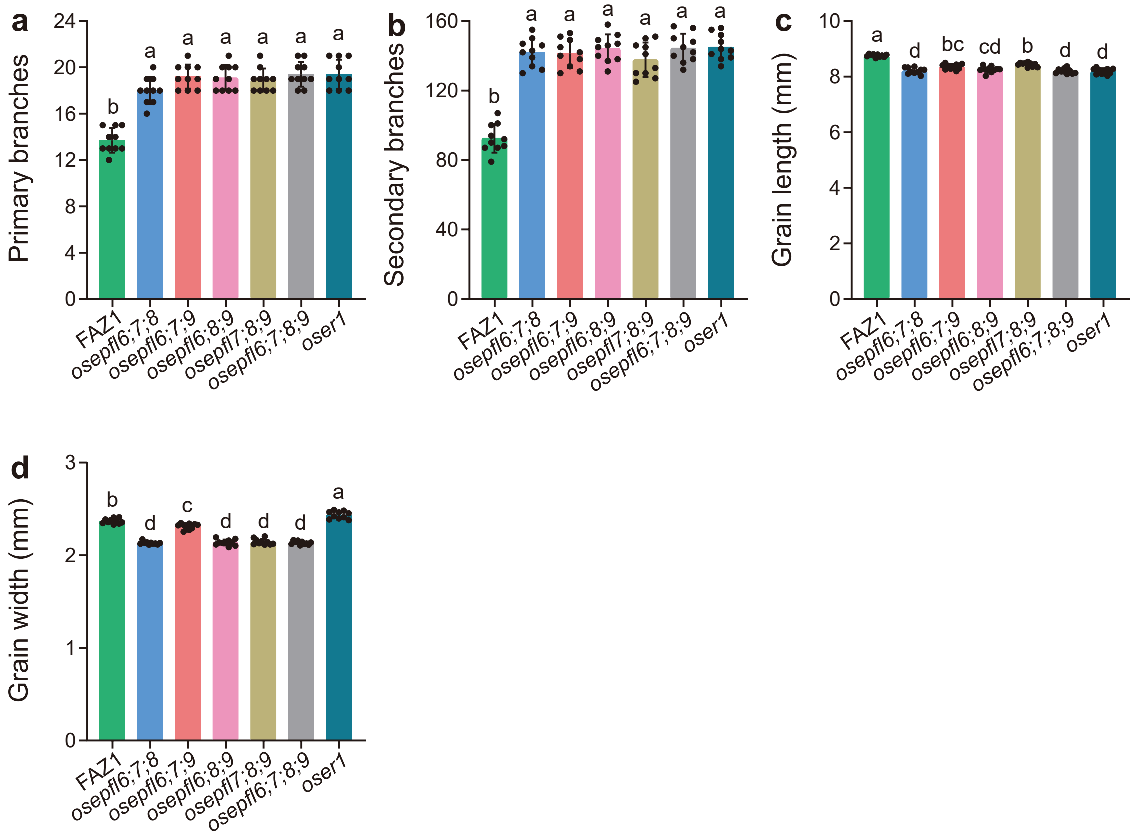

Supplementary Fig. 14 Number of primary and secondary branches, and grain size of *OsEPFL6*, *OsEPFL7*, *OsEPFL8*, and *OsEPFL9* triple and quadruple mutants.
a-d, Comparison of average number of primary branches (**a**), number of secondary branches (**b**), grain length (**c**), grain width (**d**) between FAZ1, *osepfl6;7;8*, *osepfl6;7;9*, *osepfl6;8;9*, *osepfl7;8;9*, *osepfl6;7;8;9*, and *oser1*. Values are given as the mean ± SD (n = 10 plants). Different letters indicate statistical significance groups at $p < 0.05$ (one-way ANOVA with post-hoc Tukey's multiple comparison test). The source data underlying the statistical analysis in **a-d** are provided in the Source Data file.

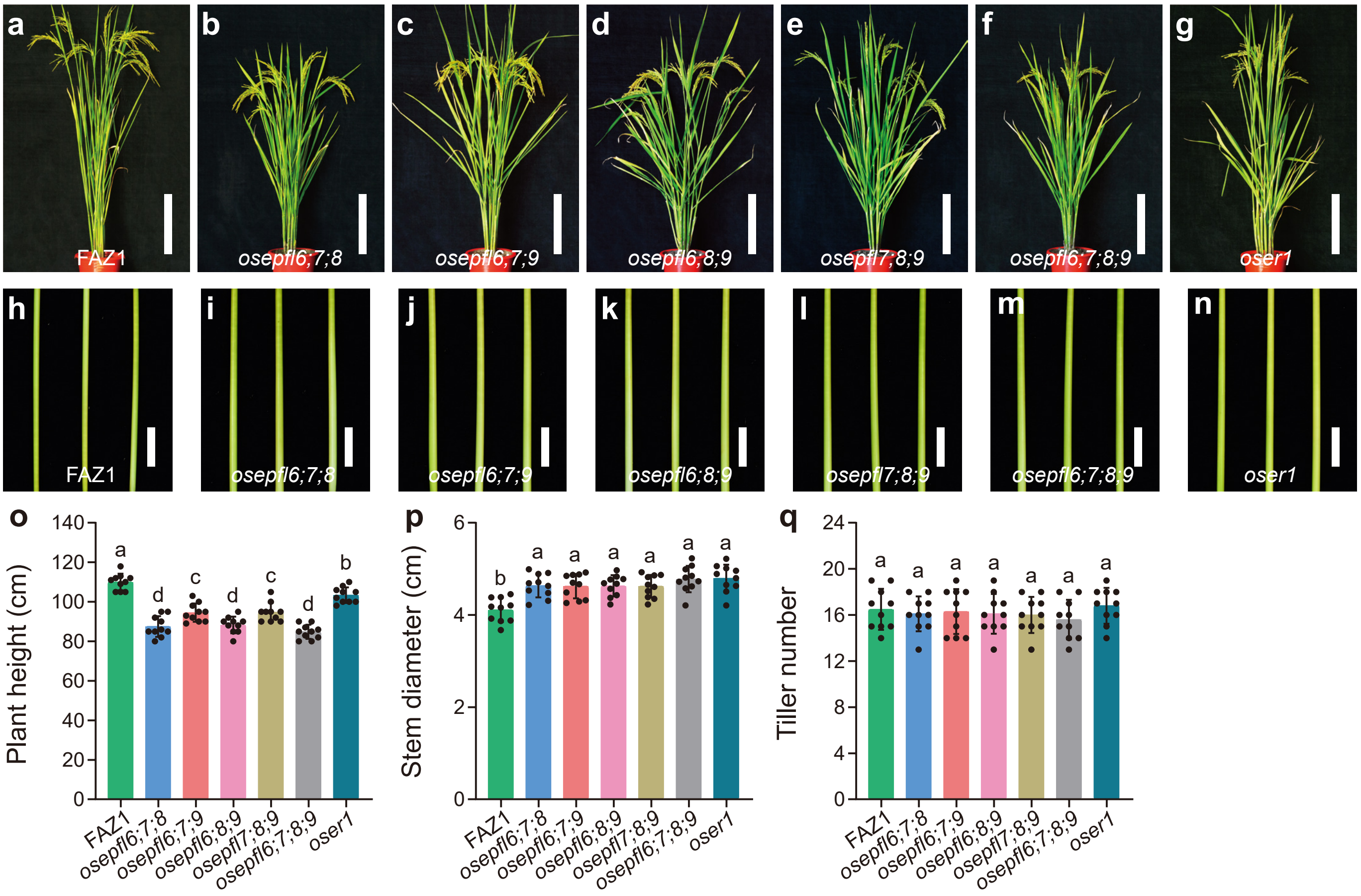

Supplementary Fig. 15 Plant architecture of *OsEPFL6*, *OsEPFL7*, *OsEPFL8*, and *OsEPFL9* triple and quadruple mutants.

a-g, Plant architecture of FAZ1 (**a**), *osepfl6;7;8* (**b**), *osepfl6;7;9* (**c**), *osepfl6;8;9* (**d**), *osepfl7;8;9* (**e**), *osepfl6;7;8;9* (**f**), and *oser1* (**g**) plants. Scale bar = 20 cm. **h-n**, Stems of FAZ1 (**h**), *osepfl6;7;8* (**i**), *osepfl6;7;9* (**j**), *osepfl6;8;9* (**k**), *osepfl7;8;9* (**l**), *osepfl6;7;8;9* (**m**), and *oser1* (**n**) plants. Scale bar = 2 cm. **o-r**, Comparison of average plant height (**o**), stem diameter (**p**), and tiller number (**q**) between FAZ1, *osepfl6;7;8*, *osepfl6;7;9*, *osepfl6;8;9*, *osepfl7;8;9*, *osepfl6;7;8;9*, and *oser1*. Values are shown as the mean ± SD (n = 10 plants). Different letters indicate statistical significance groups at $p < 0.05$ (one-way ANOVA with post-hoc Tukey's multiple comparison test). The source data underlying the statistical analysis in **o-q** are provided in the Source Data file.

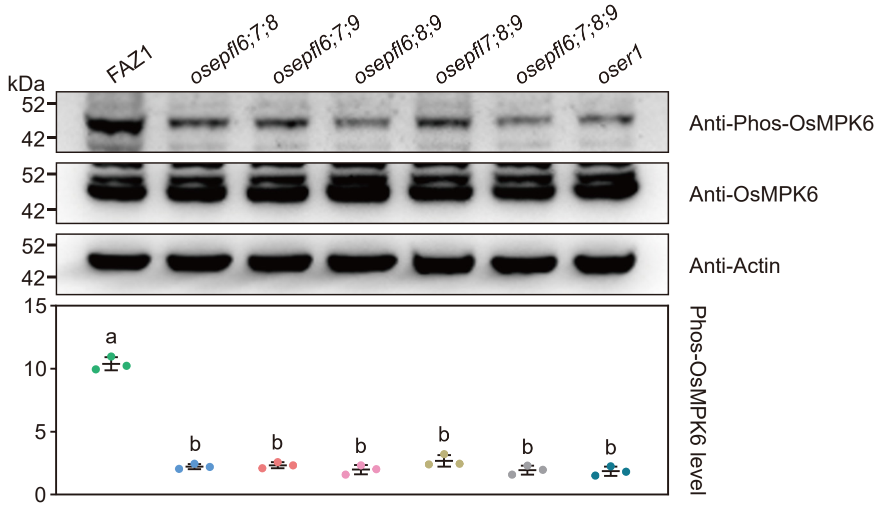

Supplementary Fig. 16 OsMPK6 phosphorylation levels were markedly reduced in the *OsEPFL6*, *OsEPFL7*, *OsEPFL8*, and *OsEPFL9* triple and quadruple mutants. Comparison of OsMPK6 phosphorylation levels in FAZ1, *osepfl6;7;8*, *osepfl6;7;9*, *osepfl6;8;9*, *osepfl7;8;9*, *osepfl6;7;8;9*, and *oser1*. Proteins extracted from young panicles were analyzed with immunoblot using anti-Phos-OsMPK6 and anti-OsMPK6 antibodies. Anti-Actin antibody was used as the loading control. The graph shows the qualification of the relative levels of the phosphorylated OsMPK6. Values are given as the mean ± SD (n = 3 biological replicates). Different letters indicate statistical significance groups at $p < 0.05$ (one-way ANOVA with post-hoc Tukey's multiple comparison test). The source data underlying the uncropped image and statistical analysis are provided in the Source Data file.
