# Supplementary material for: Optimization of rice panicle architecture by specifically suppressing ligand–receptor pairs
Source: Nat Commun. 2023 Mar 24;14:1640. doi: 10.1038/s41467-023-37326-x (PMC10039049; doi:10.1038/s41467-023-37326-x)
Supplement: Supplementary file 3 — Description of Additional Supplementary Files [file 41467_2023_37326_MOESM3_ESM.pdf]

Description of Additional Supplementary Files:

Supplementary Data 1: Primers used for CRISPR/Cas9 gene editing, and gene cloning and vector construction for overexpression, eukaryotic expression and expression pattern analysis.
